# Effect of Substrate symmetry on the dendrite morphology of MoS_2_ Film synthesized by CVD

**DOI:** 10.1038/s41598-017-13238-x

**Published:** 2017-11-09

**Authors:** Di Wu, Tai Min, Jian Zhou, Chen Li, Guobin Ma, Gaotian Lu, Minggang Xia, Zhengbin Gu

**Affiliations:** 1 Center of Spintronics and Quantum System, State Key Laboratory for Mechanical Behavior of Materials, School of Material Science and Engineering, Xian Jiaotong University, Xi’an, 710049 China; 2 National Laboratory of Solid State Microstructures and Department of Materials Science and Engineering, Nanjing University, Nanjing, 210093 China; 3 Department of Applied Physics, School of Science, Xi’an Jiaotong University, Xi’an, 710049 China; 4 Department of Optical Information Science and Technology, School of Science, Xi’an Jiaotong University, Xi’an, 710049 China

## Abstract

In van der Waals epitaxial growth, the substrate plays a particularly important role in the crystal morphology. Here, we synthesized MoS_2_ by chemical vapour deposition on silicon carbide (SiC). The obtained MoS_2_ dendritic crystals show six-fold symmetry, which are different from the conventional triangular shapes on SiO_2_ substrate and from those with three-fold symmetry on SrTiO_3_ substrate. Interestingly, these MoS_2_ dendritic crystals on SiC exhibit an average fractal dimension 1.76, which is slightly larger than the classical Diffusion-limited-Aggregation fractal dimension 1.66. The first principle calculation indicates that the six-fold symmetry of the dendritic MoS_2_ is determined by the lattice symmetry of SiC. To further demonstrating the substrate effect, we break the natural six-fold lattice symmetry of SiC (0001) into groove arrays through etching the substrate. And then we successfully synthesized cross-type dendritic crystal MoS_2_ with two-fold symmetry. Its average fractal dimension 1.83 is slightly larger than the fractal dimension 1.76 of the previous MoS_2_ dendrite with six-fold symmetry. In a word, the symmetry of SiC substrate determined the symmetry and the fractal dimension of the dendritic MoS_2_. This work provides one possibility of inducing the growth orientation of dendritic crystals through controlling the substrate surface symmetry artificially.

## Introduction

In the past ten years, two dimensional (2D) transition-metal dichalcogenides (TMDs) materials have received considerable research interests in many fields for their wide range of potential applications, including optoelectronic devices^1–3^, capacitors^4^, hydrogen storage^5^, catalysis^6^, and solid lubricant^7^, because of their unique electrical, chemical and mechanical properties. Among many transition-metal dichalcogenides, one of the most promising representatives is molybdenum disulfide (MoS_2_). It has been expected as a candidate of flexible small quantum transistor materials^8,9^ in post-Moore era, due to its moderate direct band gap at few layers^10–12^ comparing to semi-metallic graphene. Until now, a variety of methods have been developed^13–16^ to fabricate a few layers or monolayer MoS_2_, including mechanically exfoliation, liquid exfoliation, thermal decomposition and chemical vapour deposition (CVD). Among them, the CVD method has been demonstrated as a promising method in producing large continuous MoS_2_ films^17^, thickness modulated MoS_2_ films^18,19^, and even large-area and highly quality monolayer MoS_2_ films^20^. In the CVD growth process, temperature, gas flow and substrate all play essential roles in determining the final film morphology, crystalline structure and quality of film. In the previous reports, the different substrates, such as amorphous SiO_2_/Si^18,21,22^, single-crystal mica^19^ and sapphire^23^, were adopted in MoS_2_ CVD growth. In these cases, the triangular MoS_2_ films were mainly synthesized due to the little influence of substrates. The synthesis of MoS_2_ film belongs to Van der Waals epitaxial growth and may be influenced by the symmetry of substrates. Such as, SrTiO_3_ with quartic symmetry, has been used to synthesize MoS_2_ dendritic flakes with three-fold symmetry^6,24,25^. In this regard, it is imperative to know the effect of substrate symmetry in MoS_2_ CVD growth process.

Though there is a paper about MoS_2_ synthesis by use of silicon carbide (SiC) as substrate, the MoS_2_ is a vertically standing triangles, and isn’t the dendritic morphology^26^. Here, we firstly synthesize dendritic MoS_2_ with six-fold symmetry and two-fold symmetry by use of single crystal SiC as a substrate, which is also different from that with three-fold symmetry on the SrTiO_3_^6,24,25^. To explain the novel morphology formation, we adopt the diffusion limited aggregation (DLA) to reveal the reaction process of initial crystal growth. It has been observed that the symmetry of the single crystal SiC substrate played an irreplaceable role in dendritic crystal formation. In order to show the dominant role of the substrate symmetry further, we etched the SiC substrate into the grooves to break the substrate original six-fold symmetry artificially. This resulted in the cross-shaped dendritic MoS_2_ films, which is different from six-fold symmetry previously. This work provides not only one possibility of artificial induction of crystal growth orientation through controlling the substrate symmetry, but also provides a case for deeply understanding the detailed dynamics in non-equilibrium crystal growth of two-dimensional materials.

## Results and Discussion

**Figure 1:**
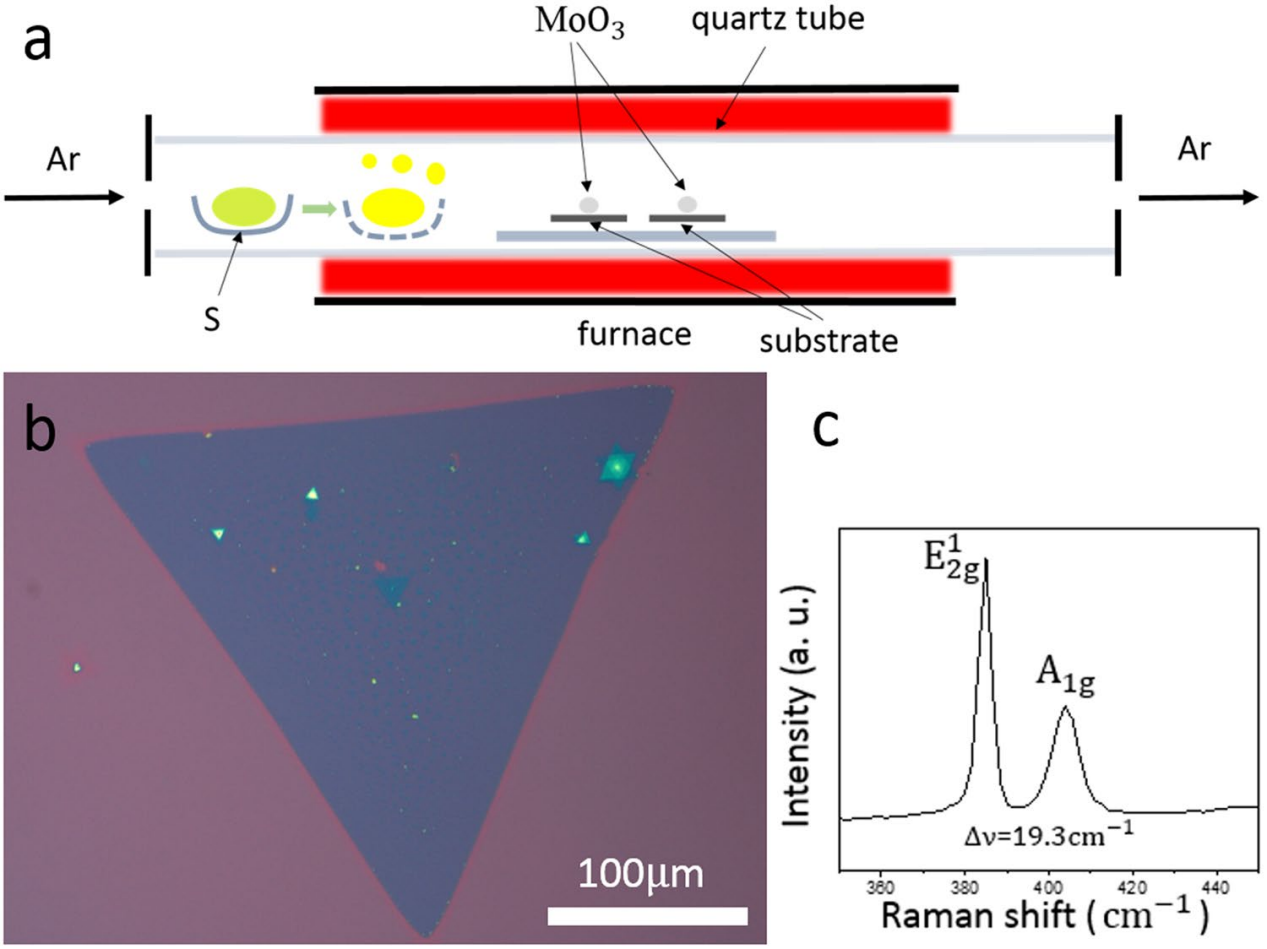
(**a**) The corresponding locations of sulphur, MoO_3_, and substrate are indicated in a single-temperature-zone CVD system. (**b**) Optical image of MoS_2_ triangle film on SiO_2_ substrate. (**c**) Relative Raman peak intensities and peak positions of the E^1^_2g_ and A_1g_ modes of the MoS_2_ triangle.

Before researching the substrate effect on film growth properties in MoS_2_ CVD synthesis, we used the common silicon substrate with 285 nm insulating SiO_2_ as a monitor of growth conditions. Through optimizing the growth conditions, ~300 μm triangular MoS_2_ crystal (Fig. 1b) has been achieved in a CVD setup (Fig. 1a) at atmospheric pressure. Raman spectroscopy was performed on this sample and relative Raman peak intensities and peak positions of the E^1^_2g_ and A_1g_ modes of the MoS_2_ have been acquired. As shown in Fig. 1c, this MoS_2_ crystal grown on SiO_2_ shows the frequency difference (∆ν) of 19.3 cm^−1^, less than 20 cm^−1^, which is a monolayer in nature^27–30^. Additionally, many secondary nucleation sites on top of the big triangle monolayer film can be easily observed, which is similar to other reports^31^. However, their formation mechanism in the final film morphology is beyond this paper’s scope.

**Figure 2:**
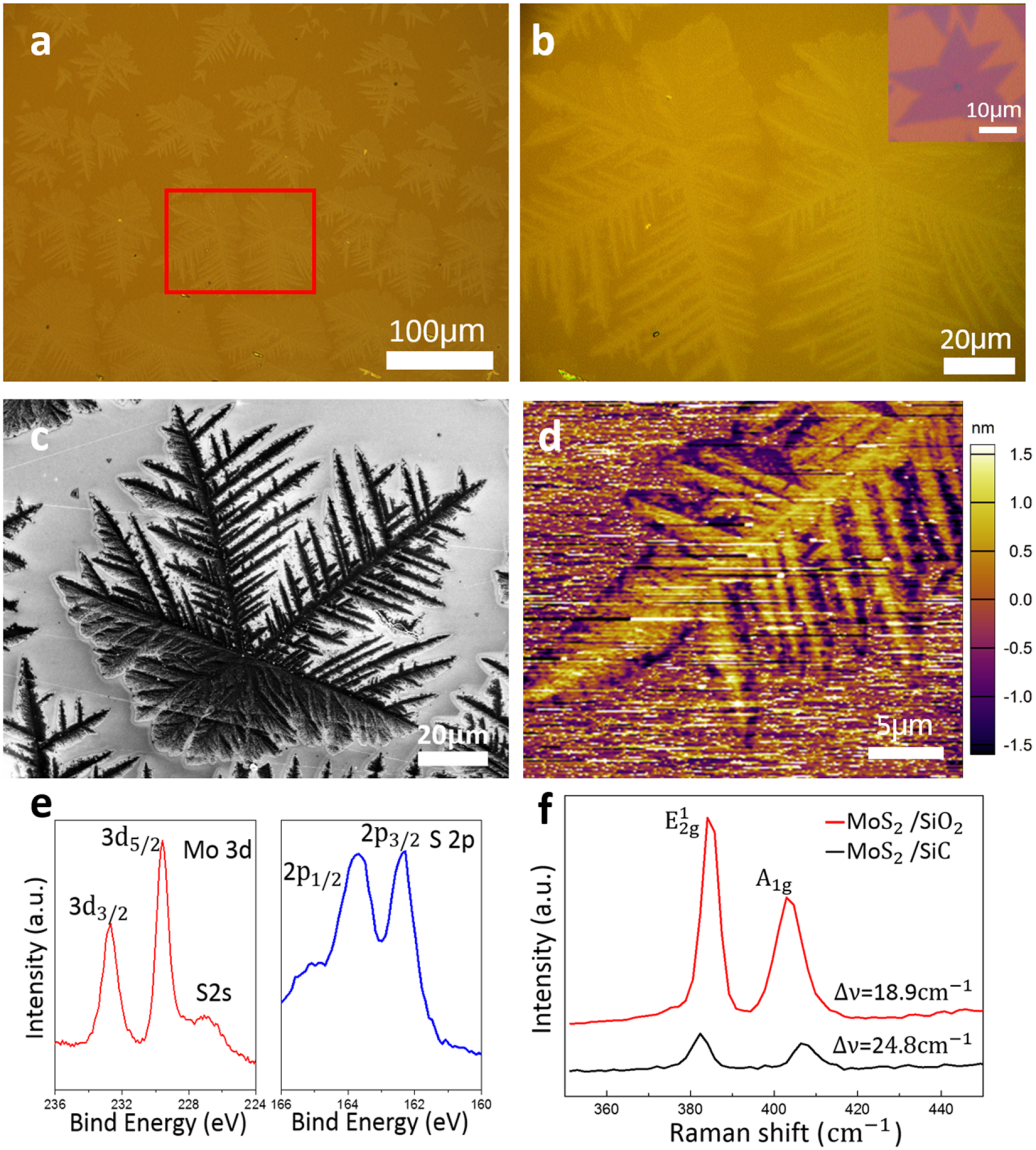
CVD synthesis, XPS and Raman characterizations of dendritic MoS_2_ on SiC. (**a**,**b**) Optics images in different amplification showing the dendritic MoS_2_ flakes grown on SiC. The inset in (**b**) is the MoS_2_ flakes grown on SiO_2_ substrate of the same batch growth as a comparison. (**c**,**d**) SEM and AFM images revealing the details of snow-like MoS_2_ flakes. (**e**) X-ray photoemission spectroscopy (XPS) data of MoS_2_ on SiC. (**f**) Raman spectroscopy data of MoS_2_ on SiC and SiO_2_.

The MoS_2_ crystal grown on SiC substrate has shown clearly dendritic form as shown in Fig. 2, instead of compact triangular structure obtained on SiO_2_/Si substrate. The optical image recorded over a large region with plentiful MoS_2_ dendritic crystal, like snowflakes, on the SiC substrate, as shown in Fig. 2a. Most of the islands’ sizes are around 100 μm. The area within the red rectangular region of Fig. 2a was zoomed in and shown in Fig. 2b. In the image, it can be observed clearly that two hexagonal dendritic crystals have same six-fold symmetric backbones, extending with many hierarchical branches harbouring similar angle. In some regions on SiC wafer, the backbones only extend a few secondary branches, while in other regions, the continuous films were observed, which may be due to different local growth conditions. The inset in Fig. 2b is the MoS_2_ flakes grown on SiO_2_ substrate in the same batch growth as a comparison. The scale bar is 10 μm. The X-ray photoelectron spectroscopy (XPS) data of the dendritic crystals grown on SiC reveals the occurrence of Mo 3d^3/2^ and 3d^5/2^ states at binding energies of 232.7 eV and 229.6 eV, and 2p^1/2^ and 2p^3/2^ states of S at 163.7 eV and 162.4 eV respectively (Fig. 2e). These are typical signs for MoS_2_ films^32–34^. To characterize the flakes’ structure in more details, a high resolution scanning electron microscope (SEM) image was taken in Fig. 2c. Similar to the optical images, it also shows the backbones and many hierarchical branches. While, there is no any hierarchical branch in some backbones. From the morphology comparison, we can found clearly that the MoS_2_ grown on SiO_2_ in the same batch growth is the compact triangle crystal, which suggests that the substrate affects the final film morphology of MoS_2_. Even though various shapes with or without additional branches can be observed, all of them exhibit the basic triangle shapes or their combining forms, which is similar to previous reports of MoS_2_ grown on SiO_2_/Si substrate^20,21,35^.

Micro-Raman spectroscopy has been widely utilized to characterize the 2D materials. The native MoS_2_ grown on SiC and SiO_2_ was scanned by 518 nm wavelength laser, and their Raman spectra were shown in Fig. 2f. It can be observed that two samples grown on SiC and SiO_2_ all show typical characteristic Raman modes of MoS_2_, E^1^_2g_ and A_1g_ peaks, around 380 and 402 cm^−1^. Their frequency difference (∆ν) for the MoS_2_ on SiO_2_ is 18.9 cm^−1^ which is a monolayer in nature^27–30^. But their frequency difference is 24.8 cm^−1^ in MoS_2_ on SiC, corresponding to a thicker film. The Atomic Force Microscope (AFM) image (Fig. 2d) reveals more details about the morphology and thickness of the snow-like MoS_2_ dendrite. It indicates that the thickness of the dendritic MoS_2_ flakes is at most 3 nm, approximate 5 layers.

**Figure 3:**
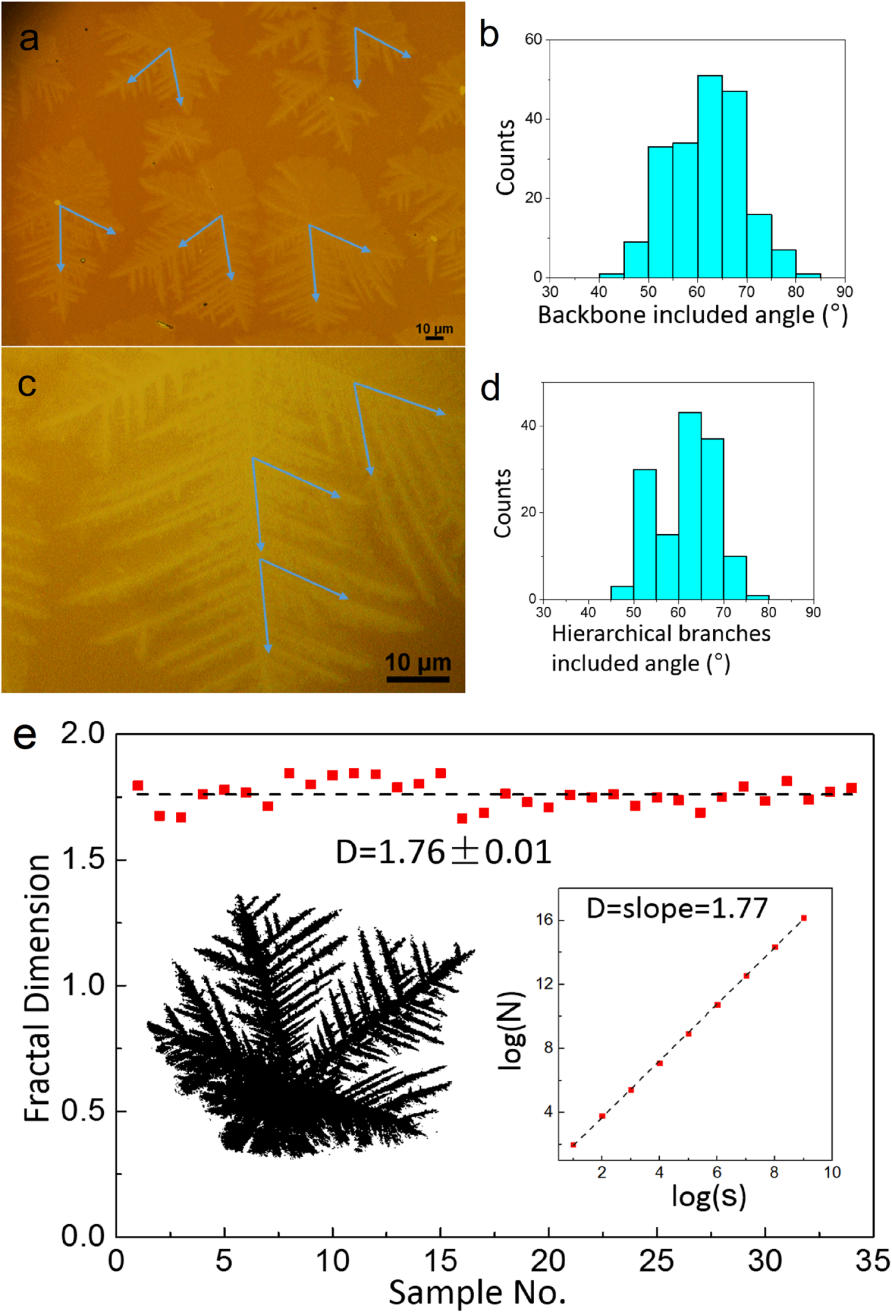
Statistics of the backbones and the hierarchical branches angles, and Fractal dimension calculation of MoS_2_ dendrites on SiC (0001). (**a**) Optical image of the MoS_2_ dendrites featured with 60° included angles backbones (highlighted with blue arrows). (**b**) Statistics of the two backbone included angles for 200 flakes. (**c**) Magnified optical image of hierarchical branches showing with 60° included angles. (**d**) Statistics of hierarchical branches included angles for 140 flakes. (**e**) Statistics for 34 snow-like MoS_2_ dendrites (Left inset: contrast-enhanced SEM image of a typical snow-like MoS_2_ dendrite; Right inset: box-counting estimation of the fractal dimension for the left flake).

The two-dimensional crystal growth mechanism of MoS_2_ can usually be described by following process: 1) nucleation, 2) diffusion and growth, 3) formation of crystals. The symmetry of substrate dominates the nucleation process and plays a critical role in determining whether a compact or fractal growth. In other words, the substrate with triangular or hexagonal geometry is easier to have extended fractal growth than square lattice^36–38^. To obtain the symmetry of the MoS_2_ film morphology in more detail via CVD growth method, we carried out the angular distribution of the dendritic morphology of MoS_2_ on SiC statistically. The orientation of backbones of dendrites were illustrated by the blue arrows, between which are 60° angles usually, shown in Fig. 3a. And the angle between the subsequent hierarchical branches and the main backbones is also about 60°, seen in Fig. 3c. It has been confirmed that both angle distributions are centred at 60°, shown in Figs 3b and d, by the angle statistics from 200 flakes of backbone and 140 flakes of hierarchical branches.

In order to describe the morphology of MoS_2_ flakes in more detail, the fractal dimension is calculated statistically, presented in Fig. 3e. Thirty-four flakes on the same sample was comprehensively estimated by use of a standard box-counting evaluation. The left inset is a contrast-enhanced SEM image of a typical snow-like MoS_2_ dendrite crystal; while the right inset is a box-counting estimation of the fractal dimension for the left flake. These flakes exhibit an average fractal dimension of 1.76 ± 0.01. This fractal dimension is in good agreement with the classical DLA dimension of 1.66. This also demonstrates the natural fractal property of the snow-like MoS_2_ dendrite indirectly.

**Figure 4:**
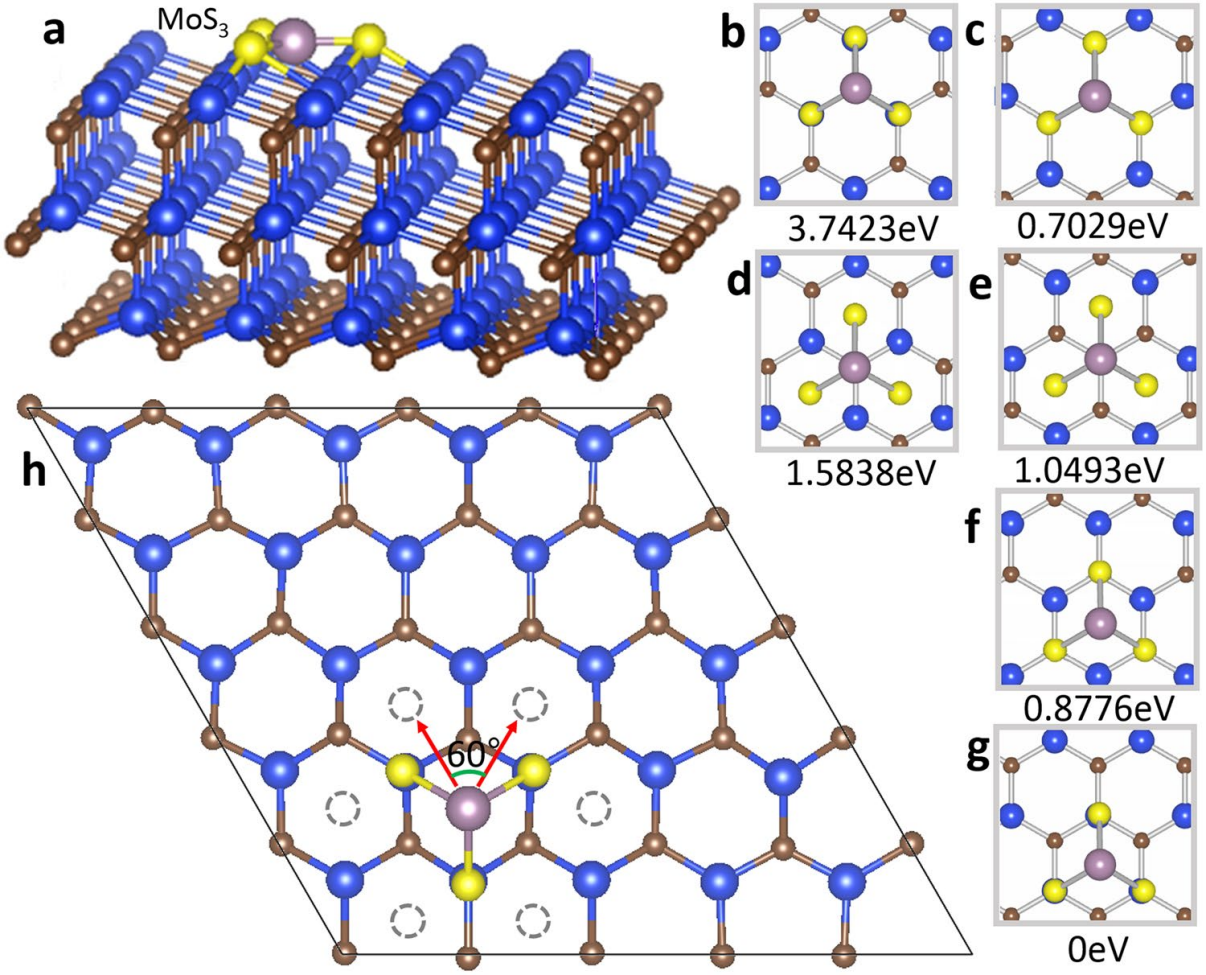
Density functional theory (DFT) calculations of assumed MoS_3_ monomer precursor on Si-terminated SiC surface. (**a**) Actual calculation unit cell with a MoS_3_ molecule and 10 Å vacuum space along the z direction. Blue, brown, light brown, and yellow balls denote the Si, C, Mo and S atoms, respectively. (**b**–**g**) Six modes of MoS_3_ molecule adopted on different position on SiC surface. Corresponding negative bonding energy were showed under the images respectively. (**h**) The most stable adsorption configuration (Fig. 4g) for a MoS_3_ monomer on SiC (0001). The red arrow reveals schematic illustration of the probable diffusion pathways for a MoS_3_ monomer on SiC (0001). Dotted circles represent the six closet adsorption sites.

Whether the six-fold symmetry observed in the dendritic morphology is influenced by the substrate surface symmetry or not? In order to reply the question, the density functional theory (DFT) calculations were performed. The binding energies of a MoS_*x*_ monomer on various configurations on the Si-terminated SiC (0001) surface are computed. Here the molecular MoS_3_ was assumed to be the representative monomer precursor for the final MoS_2_ film because it is the dominate phase in the low-temperature and excess sulphur regime, according to the Mo-S phase diagram^39^. In the DFT model, the SiC substrate with Si-terminated surface was modelled by a three layers 5 × 5 superlattice with total 150 atoms as shown in Fig. 4a. And a vacuum space of 10 Å along the direction perpendicular to the surface was used to avoid interactions between adjacent layers. Six absorption sites of MoS_3_ molecule on SiC surface were calculated and the corresponding bonding energy to the lowest energy mode (Fig. 4g) were shown under each image, respectively (Fig. 4b–g). Note that the most energetically favourable adsorption site for MoS_3_ is for the Mo atom sitting above the centre of the hexagonal lattice while the S atom locating on top of Si atoms, as shown in Fig. 4g, where the unsaturated S and Si atoms could form effective bonding with each other. Because of the natural hexagonal symmetry of SiC, it is obvious that there are six closest equal adsorption sites near the energetically favourable adsorption site and six preferential equal diffusion pathways oriented 60 degree to each other as shown in Fig. 4h. This is in agreement well with the statistical results about the 60 degree angle between the backbones of dendritic MoS_2_ flakes.

The formation of the dendritic morphology could be inferred by the DLA mechanism^36,40,41^. When the MoS_3_ molecule was adsorbed onto substrate, it might have some energy to move around on the surface. According to our calculation, the molecule prefer the position energetically on where the Mo atom is rest upon the centre of the hexagonal lattice because this configuration has the lowest binding energy, and thus it serves as an nucleation site. This nucleation on SiC substrate doesn’t like the previous SiO_2_, sapphire and Mica substrate, on which it prefers to happen on impurity or defects.

Subsequent adsorbed atoms (ad-atoms) deposit onto the substrate and diffuse in a certain way. They might hopping around onto different sites but the configuration with Mo sitting at the centre of Si-C hexagon is energetically favoured and will serve with stronger sticking coefficient. When the ad-atoms encounter those nucleation centres, three situations might happen, which is determined by the difference between particle diffusing and bonding energy. The first is that the particles don’t diffuse and become attached to the nucleation centre and being part of cluster. The second is that the ad-atoms continue walking on the substrate surface and not adsorbed with any cluster. The third is that they become one part of the cluster, but continue to diffuse along the cluster edges. With the increasing of the cluster size, more and more ad-atoms have the chance to meet the island and the cluster continues to grow. It suggests that the first and the second situations would always happen until there are no ad-atom left on the surface. What is interesting is the third case, the ad-atoms with proper energy diffuse limitedly along the edge so that they couldn’t cross the corner to make the edge relaxing and new corners would be generated. Subsequent ad-atoms follow the former atoms and more edge are generated. With the increasing of edges and corners, the ad-atoms have less chance to fill the vacancy sites and relax along the edge^36,37^. In our case, it suggests that because the SiC substrate has strong dangling bond^42,43^, comparing to amorphous SiO_2_, the MoS_*x*_ molecule need higher energy to diffuse along the edge so that it prefers to stick to the nucleation sites, according to the DFT calculation, leading to more edges and insufficient edges relaxation once they encounter a cluster. In a word, whether precursors could diffuse freely or form dendrite in the substrate surface depends on the competition among its energy, the temperature, the interaction between the precursor and the substrate, and the interaction between the precursor and the established island or cluster. In our case, since the strong dangling bond of the SiC substrate, the more compact dendritic MoS_2_ film was formed and its fractal dimension is bigger than the classical one. Furthermore, due to the natural hexagonal symmetry of those nucleation sites on SiC (0001) surface, the dendritic edges prefer arranging along the centre of hexagonal Si-C ring to form a unique dendritic shape with six-fold symmetry. About the dendritic shape with six-fold symmetry in two dimension, there is another material, graphene dendritic on copper^44^. MoS_2_ dendritic with six-fold symmetry is different from the graphene one because the former owns three atoms layer structure, while the latter is only one atom layer structure. The most important is that the morphology of graphene dendritic is determined by itself symmetry, not by the substrate, copper. MoS_2_ dendritic is fully determined by the symmetry of the SiC substrate^44^. Furthermore, from DLA mechanism, we can know that another energy term in first principle calculation, a stick potential barrier works in MoS_2_ dendritic growth.

**Figure 5:**
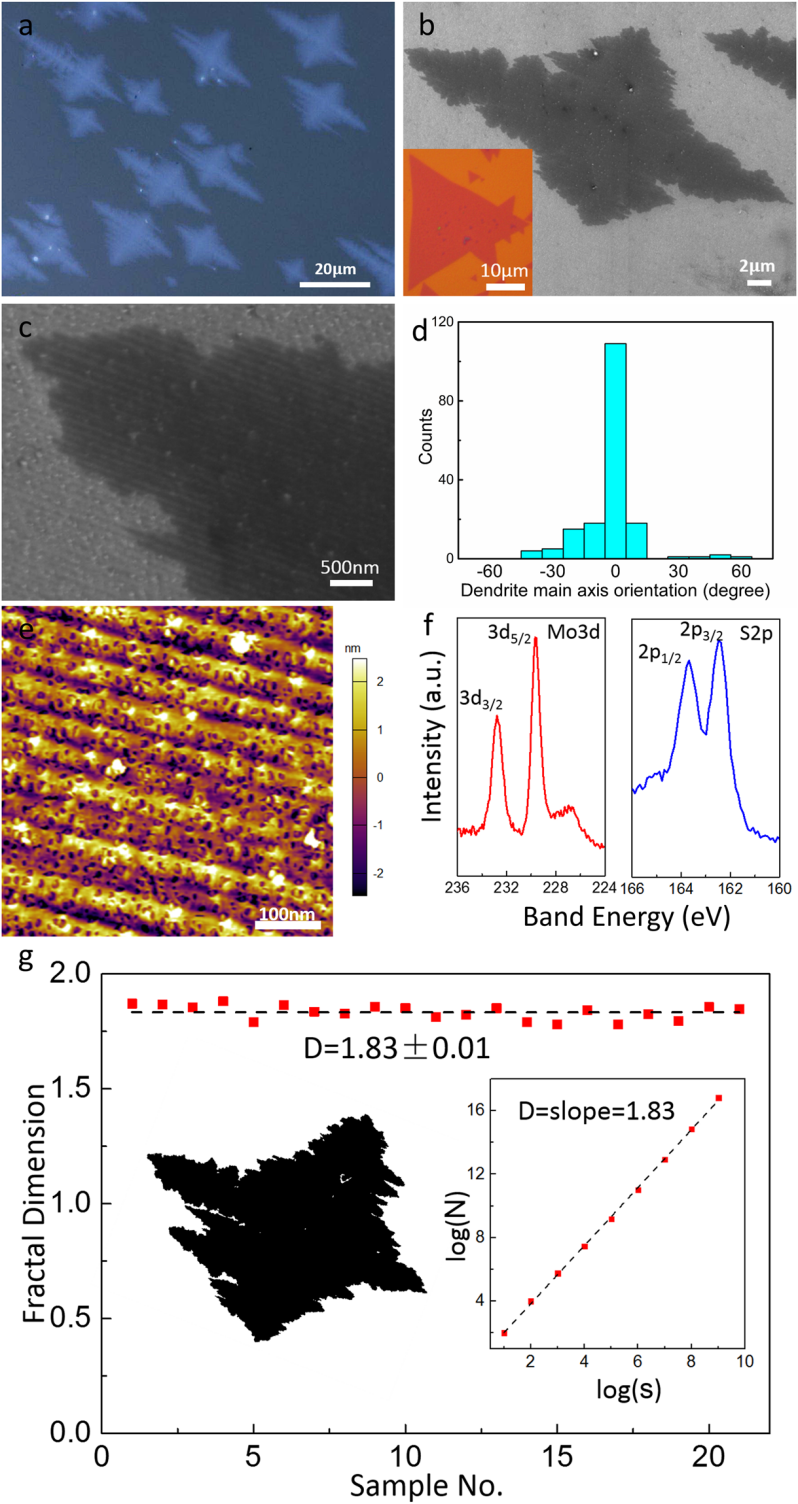
CVD synthesis and Fractal dimension calculation of MoS_2_ on etched SiC and SiO_2_. (**a**–**c**) Optics and SEM images show the cross-type MoS_2_ flakes on etched SiC in different magnification. The inset in (**b**) shows the MoS_2_ flakes grown on SiO_2_ substrate in the identical furnace as comparison. (**c**) SEM images showing the MoS_2_ polycrystalline on etched SiC in a different scale. The inset is the left partial enlarged view of the dendritic flake. (**d**) Statistics of the dendrite main axis orientation angles for 140 flakes. (**e**) The AFM image showed the groove of MoS_2_ on etched SiC. (**f**) XPS data of MoS_2_ on etched SiC. (**g**) Statistics for 21 cross-type dendritic MoS_2_ flakes (Left inset: contrast-enhanced SEM image of a typical cross-type MoS_2_ dendrite; Right inset: box-counting estimation of the fractal dimension for the left flake).

To further demonstrate that the SiC surface symmetry determines the final dendritic morphology of MoS_2_ film in CVD, we created an artificial surface structure on substrate to change the nucleation sites symmetry. The chemical etching process is presented to accomplish the aim. Prior to MoS_2_ growth, the SiC substrate was immersed in hydrofluoric acid (HF, 1%) for 24 h before washing. Artificial grooves with about 3 nm depth and 100 nm width was created, shown in Fig. 5e. The MoS_2_ film grown on the etched SiC shows a cross-type dendrite with two-fold symmetry, as shown by the optical and SEM images of Figs 5a–c. Most of the island’s size are around 20 μm, much smaller than 100 μm crystals on native SiC. As a comparison, the MoS_2_ on SiO_2_ under the same batch growth is shown in the inset of Fig. 5b (the scale bar is 10 μm). By comparing these images, it is obvious that the new two-fold symmetry MoS_2_ dendrite is different from the six-fold MoS_2_ on native SiC and from the compact crystal on SiO_2_. Furthermore, the orientation of the two-fold dendrites are aligned with the long axis along roughly the same direction of the grooves. This has been suggested by the statistics of main axis orientation for the dendritic flakes, seen in Fig. 5d. To confirm the crystal grown on etched SiC is still MoS_2_, XPS was also collected, and the result is shown by Fig. 5f: the occurrence of Mo 3d^3/2^ and 3d^5/2^ states at binding energies of 232.7 eV and 229.6 eV, and 2p^1/2^ and 2p^3/2^ states of S at 163.7 eV and 162.4 eV, which are typical signs for MoS_2_ film. Furthermore, the SEM and AFM images have shown more morphology details (Figs 5c,e). Wavy MoS_2_ flakes with groove arrays can be observed, which indicates again that the long axis of cross-type dendrite is along the groove direction. Comparing to the six-fold MoS_2_, the fractal dimension of two-fold MoS_2_ is around 1.83 by statistics for twenty-one cross-type dendritic MoS_2_ flakes (Fig. 5g). The fractal dimension is slightly bigger than that of the six-fold one, so we can infer the two-fold dendritic MoS_2_ is more compact than six-fold one. It is attributed to a potential barrier. On the etched SiC substrates, the precursor should overcome an additional potential barrier across artificial groove arrays to diffuse on the surface, than on the native SiC substrate.

The details of the two-fold dendrite growth should be further understood under DLA mechanism as well. On the etched SiC substrates, the artificial grooves create an anisotropy diffusion potential barrier, which induces an anisotropy sticking energy. Therefore, the energy should hinder diffusion of the precursor molecule across the groove direction, comparing to direction along the groove. During growth, the ad-atoms have much higher probability stuck to those sites at edge of grooves, causing faster growth of crystal along the groove direction than across it. Comparing the native SiC substrate, the hexagonal symmetry of the diffusion paths have been broken, which are replaced by insufficient diffusion across the grooves and faster diffusion along to the grooves. This suggests that two orthometric pathways in the etched SiC substrate lead to the novel cross-type shape dendrites under the DLA mechanism. Therefore, we conclude that the dendritic morphology of MoS_2_ grown on SiC substrate can be modified to the desired morphology through artificially constructing special substrate surface in the CVD growth process. This also inspires us to explore novel symmetry of dendrite, different from its natural, by the use of special substrate or the special symmetry structure on surface of substrate.

## Conclusion

In summary, we have achieved the MoS_2_ dendritic in CVD on single-crystal SiC. The obtained MoS_2_ flakes show a special snow-shaped dendritic morphology with six-fold symmetry, in contrast with the traditional sharp-edged triangular compact flakes grown on conventional SiO_2_ substrate. The growth dynamics of this morphology can be readily explained by the classical Diffusion-Limited-Aggregation mechanism. Additionally, we find the intrinsic lattice symmetry of the SiC substrate can be broken by etching, and obtained cross-shaped dendritic MoS_2_ films, further suggesting that the substrate plays a crucial role in dendritic growth process. Furthermore, by comparing the two kind of fractal dimension of dendrite with the classical one, we found that the substrate effect on the MoS_2_ dendrite would be enhanced due to the additional potential barrier. This work could contribute to further in-depth understanding of the detailed dynamics in non-equilibrium crystal growth of two-dimensional materials and provides one possibility of artificially inducing the growth orientation of crystals through controlling the substrate surface symmetry.

## Methods

The MoS_2_ flakes were produced utilizing the facile CVD technique. High purity MoO_3_ (99.5%, Alfa Aesar) and sulphur powder (99.9%, Alfa Aesar) were used as precursors. The MoO_3_ powder (less than 1 mg) was sowed directly on the substrate which was placed at the centre of a 4-inch diameter CVD furnace by a flat quartz board. The sulphur powder was kept in a quartz boat placed upstream at a lower temperature zone out of the furnace. The distance between sulphur boat and the substrate was maintained to be ~50 cm.

We used single crystal 4H-SiC wafers as substrates, where the crystal orientation is <0001> and 4° toward <112(−)0>. All substrates were first immersed in a freshly prepared piranha solution (H_2_SO_4_:H_2_O_2_, 7:3) for 24 h. Then they were washed with de-ionized (DI) water two times, repeatedly, and sonicated in acetone and subsequently in isopropyl alcohol (IPA) for 5 min each before blow-dried by nitrogen. In addition, when it needs etching, the SiC substrate was etched by immersing in hydrofluoric acid (HF, 1%) for 24 h. And all other processes were then completed repeatedly.

Prior to the growth, the quartz tube of CVD furnace was cleaned completely. The furnace was then heated to 300 °C for 60 min under Ar gas. Then the temperature was ramped up to 700 °C at a rate of 10 °C/min. At this stage, the sulphur boat was pushed into heated zone to a suitable position. Then the furnace was kept in 700 °C for 20 min before cooled naturally. In the whole process, the gas flow rate was kept at 500 sccm.

The SEM measurements were done using a Zeiss Ultra 55 FESEM system. The Raman spectroscopy were executed at room temperature in back scattering geometry using a Renishwa’s Invia confocal Raman spectrometer with argon ion laser (514 nm excitation). The size of the focused laser spot on the sample was ~1um. The laser power was kept at 12 μW for all the measurements. The X-ray photoelectron spectroscopy (XPS Thermo Fisher K-Alpha) measurements were done with standard Al K_α_ (1486.7eV) x-ray source. The binding energies were calibrated with respect to the signal from the adventitious carbon (binding energy =284.5eV). AFM measurement was performed on an Asylum Research Company Cypher-ES system.
